# Graham Bread

**Published:** 1878-01

**Authors:** 


GRAHAM BREAD.—Take the unbolted flour of wheat, wet it with lukewarm water, add salt and yeast, knead in enough more of this flour to make it stiff, add a little molasses, and, when risen, bake in medium-sized loaves.

ERYSIPELAS is said to be cured by applying to the part affected, a paste made of raw cranberries beaten.

KETTLES are cleansed of onion and other odors, by dissolving a teaspoonful of pearlash or saleratus in water, and washing them.

HAIR, removed by fevers and other sickness, is made to grow by washing the scalp with a strong decoction of sage leaves once or twice a day.

STINGS and bites are often instantaneously cured by washing them in hartshorn or turpentine.

FLIES DESTROYED.—A pint of sweet milk, a quarter of a pound of sugar, two ounces of ground pepper, simmer together for ten minutes, and place it about in shallow dishes. If this is true, there is no necessity for using poisonous articles about a house.

BOILING POTATOES.—It is said that in Ireland they always nick off a piece of the skin, put them in a pot of cold water, which is gradually heated, but never allowed to boil; cold water should be added as soon as the water begins to boil; when done, pour all the water off, cover the vessel with a cloth, and in a few minutes they are cool enough for use.

BUY the best articles for family use; for, although they cost more, good articles spend best.

SUGAR from Havana is always dirty, that from Brazil is clean, as also from Porto Rico and Santa Cruz. Refined sugars, whether loaf, crushed, or granulated, are the cheapest in the end.

LARD from a hog not over a year old, is the best, and should be hard and white.

BUTTER made in September and October is the best for winter use.

RICH cheese feels softer under the pressure of the finger. That which is very strong is neither very good nor healthy. To keep one that is cut, tie it up in a bag that will not admit flies, and hang it in a dry cool place. If mold appears on it, wipe it off with a dry cloth.

FLOUR and meal of all kinds should be kept in a cool dry place.

THE best rice is large, and has a clear fresh look. Old rice sometimes has little black insects inside the kernels.

THE small white sago, called the pearl sago, is the best. The large brown kind has an earthy taste. This article and tapioca, ground rice, etc., should be kept covered.

To select nutmegs, pick them with a pin. If they are good, the oil will instantly spread around the puncture.

KEEP coffee by itself, as the odor affects other articles. Keep tea in a close chest or canister.

ORANGES and lemons keep best wrapped close in soft paper, and laid in a drawer of linen.

THE cracked cocoa is best; but that which is put up in pound papers is often very good.

SOFT soap should be kept in a dry place in the cellar, and not be used until three months old.

To thaw frozen potatoes, put them in hot water. Frozen apples in cold water, but use them at once.

OVER-EATING.—As soon as you are sensible that you have eaten too much, take a walk, gradually increasing its rapidity until there is a free perspiration, and continue at this gait until every feeling of discomfort about the stomach or lungs has disappeared, then cool off very slowly in a closed room, and eat not an atom until the second meal thereafter, thus omitting one.

SICK headache is almost always attended with cold feet, and the failure of a daily action of the bowels; and there is no permanent cure without the rectification of these.

WHITEWASH.—White fences and outbuildings indicate the thrifty farmer and a tidy household. Put half a bushel of unslacked lime in a clean, tight barrel, pour over it boiling water until it is covered five inches, stir briskly until the lime is thoroughly slacked, then add more water until it is as thin as desired, next add two pounds of sulphate of zinc and one of common salt; then apply with a common whitewash brush, giving a good coat in April and October, or at least once a year.

If you get your feet or body wet, keep moving with sufficient briskness to keep off a feeling of chilliness until you get to the house; undress instantly by a warm fire, drinking, as soon as possible, a cup or two of hot tea of any sort, and remain by the fire until thoroughly rested.

When from any cause the bowels fail to act at the usual time, do not eat an atom more until they do act, at least for thirty-six hours; the first meal after a fast should be very light, of bread and butter, and a cup of weak tea or coffee.

Biliousness is indicated by a bad taste in the mouth of mornings, a poor appetite, and a feeling of general discomfort, often accompanied with a headache and cold feet. The best cure is to work moderately, take but two meals a day, and these of bread and butter, with a cup of tea or coffee.

Poison of almost any kind swallowed will be instantly thrown from the stomach by drinking half a glass of water, (warm is best,) in which has been stirred a tablespoon of ground mustard; as soon as vomiting ceases, drink a cup of strong coffee, into which has been stirred the white of an egg; this nullifies any remnant which the mustard might have left.

Paste may be made with flour in the usual way, but rather thicker, with a proportion of brown sugar, and a small quantity of corrosive sublimate. A drop or two of the essential oil of lavender, peppermint, anise, or bergamot, is a complete security against molding. Paste made in this manner, if kept in a close covered pot, may be preserved in a state fit for use at any time.

Liquid glue is made by dissolving a pound of common glue with heat in a pound of strong vinegar, and one quarter of a pound of alcohol; this is whitened by adding sulphate of lead.

Moths are kept from carpets by sprinkling salt and pepper, mixed in equal quantities, about and under the edges.

Bed-bugs are kept away by washing the crevices with strong salt water, put on with a brush.

Picture-frames and glasses are preserved from flies by painting them with a brush dipped in a mixture made by boiling three or four onions in a pint of water.

An ink-stand was turned over on a white table-cloth, a servant threw over it a mixture of salt and pepper plentifully, and all traces of it disappeared.

ANTS are kept out of drawers and other places by spirits of camphor.

BUTTER KEPT FRESH.—Take it as it comes from the churn, and wash the butter-milk thoroughly out of it, then dry the surface of the butter with a clean cloth, break into small pieces, and pack it solid into a crock. The air must be entirely expelled. Set the crock in a kettle half-filled with water, then place the kettle over the fire until the water boils. While boiling remove from the fire, and let the crock remain in the water until cold.

A MEDICINE.—Abernethy's prescription to a wealthy patient was: "Let your servant bring you three or four pails of water, and put it into a wash-tub; take off your clothes, get into it, and from head to foot rub yourself well with it, and you'll recover."

"This advice of yours seems very much like telling me to wash myself," said the patient.

"Well," said Abernethy, "it is open to that objection."

A PRESCRIPTION.—If you wake up thirsty, diet, that is, eat nothing; if you have diarrhea, be quiet, that is, do nothing, drink nothing; and if not better in twelve hours, send for a physician.

LIGHTNING-RODS, in cities, says Prof. Henry, of the Smithsonian Institute, should be connected with the water or gas-pipes underground, outside the building.

A BIT of glue dissolved in skim-milk and water, will restore old crape. Half a cranberry bound on a corn will soon kill it.

RED ANTS.—Wash your shelves down clean, and while damp rub fine salt on them quite thick, and let it remain on for a time, and they will disappear.

EYES.—If you must sew on black cloth at night, pin a piece of soft white paper along the seam, and sew through it; afterwards tear the paper away.

CAMOMILE.—The decoction of its leaves is said to destroy various insects; the living plant imparts health to other plants, often reviving drooping ones.

POTATOES contain nearly all their nutriment (the starch) very near the surface; the heart has but little; hence, let the peeling be the thinnest possible.

